# Exploring the determinants of risk behavior for transfusion transmissible infections among first-time blood donors in Mandalay General Hospital, Myanmar

**DOI:** 10.1371/journal.pone.0304134

**Published:** 2024-05-23

**Authors:** Myo Zin Oo, Soe Sandi Tint, Nutchar Wiwatkunupakarn, Alessio Panza, Chaisiri Angkurawaranon, Kyaw Min Oo

**Affiliations:** 1 Department of Family Medicine, Faculty of Medicine, Chiang Mai University, Chiang Mai, Thailand; 2 Global Health and Chronic Conditions Research Center, Chiang Mai University, Chiang Mai, Thailand; 3 College of Public Health Sciences, Chulalongkorn University, Bangkok, Thailand; 4 Department of Preventive and Social Medicine, University of Medicine, Mandalay, Myanmar; University of Health and Allied Sciences, GHANA

## Abstract

**Introduction:**

Blood donation is vital to healthcare, but it must be kept safe by mitigating the risk of transfusion transmissible infections (TTIs). The objective of this study was to investigate the factors that influence risk behavior for transfusion transmissible infections among first-time blood donors at Mandalay General Hospital, Myanmar.

**Methods:**

This study utilized a cross-sectional study design using secondary data. Mandalay city and Mandalay Blood Bank in Mandalay General Hospital were purposely selected and a total of 406 first-time blood donors participated. A structured questionnaire administered by an interviewer was used. The questionnaire contained background characteristics, knowledge on TTIs, attitude toward TTIs, and TTIs risk behaviors. To examine the determinants (background characteristics, knowledge, and attitude) that affect risk behavior, inferential statistics techniques that included the chi-squared test, bivariable logistic regression, and multivariable logistic regression were applied. A p-value of less than 0.05 signified statistical significance.

**Results:**

Among 406 first-time blood donors, 52.9% were under 20 years old, and 53.7% were male. Most had undergraduate education (77.6%), were married (84.2%), and were students (55.7%). Additionally, 76.8% hadn’t received the hepatitis B vaccine. Blood groups were distributed as follows: B (40.0%), O (33.8%), A (23.4%), AB (8.9%). About 15.8% showed high knowledge level, and 63.6% had high attitude. Notably, 29.3% exhibited high-risk behavior for TTIs. Age was associated with lower risk behavior (OR = 1.54, 95% CI: 0.99, 2.38, *p* = 0.049), but lost significance in multivariable regression (*p* = 0.214). Knowledge on TTIs didn’t show significance. However, high attitudes were significantly associated with lower risk behavior (OR = 11.4, 95% CI: 1.25, 103.83, *p* = 0.017, retained in multivariable regression, *p* = 0.012).

**Conclusion:**

Findings of this study contribute in the development of programs that ensure a safe and reliable blood supply chain. To improve blood safety standards among first-time blood donors, this study highlights the value of targeted education and screening processes, placing particular emphasis on acquiring knowledge and positive attitude toward blood donation and risk behavior.

## Introduction

Millions of lives have been saved by blood donation all around the world. The World Health Organization (WHO) recognizes safe blood access as essential human rights [1]. According to WHO, children under the age of 5 receive 54% of blood transfusions in low-income countries, while individuals aged 60 and above account for 76% of transfusions in high-income countries [2].

Infections called transfusion transmissible infections (TTIs) develop when a person receives a blood transfusion while harboring a pathogen [3]. TTIs such as human immunodeficiency virus (HIV), syphilis, hepatitis B virus (HBV), and hepatitis C virus (HCV) exert a considerable adverse impact on global public health [4]. High-income countries exhibit significantly lower rates of TTIs in blood donors compared to low- and middle-income countries [1, 5]. The WHO guideline states that every donated blood must go through a rigorous infection screening process before it is allowed for use [1]. Syphilis, HBV, HCV, and HIV testing are required as part of this screening process.

In blood donation, knowledge and attitude have played an important part [6]. Prevention of TTIs depends critically on understanding of the knowledge, attitude, and risk behavior of first-time blood donors. Blood donor screening questionnaires, which are crucial for determining health status and infection risk, may have limitations due to the fact that some donors may not completely disclose deferrable risk behavior as a result of the socially sensitive nature of questions [7]. By recognizing high-risk behavior, early intervention and counseling can be provided, to safeguard the blood supply [8].

According to the authors’ present knowledge, this study is the first to examine the risk behavior of TTIs among first-time blood donors in Myanmar, addressing its factors which are knowledge and attitude. The development of tailored treatments and interventions to reduce the spread of infections through blood transfusions in Myanmar can be made possible by the information on a thorough understanding of the factors that influence risky behavior. This present study was aimed to explore the determinants of risk behavior for TTIs among first-time blood donors at Mandalay General Hospital, Myanmar.

## Materials and methods

### Study design and data source

This present study applied a cross-sectional analytical study design using secondary data from the Department of Preventive and Social Medicine, University of Medicine, Mandalay. The department surveyed first-time blood donors from April to July 2016 regarding their demographic and risk behavior with regard to TTIs.

### Study area and study setting

Mandalay General Hospital was purposively chosen. With a population of 5.76 million in 2011, the Mandalay region, which includes Mandalay as its capital, is the third-largest region in the country [9]. The largest and primary teaching hospital in Mandalay, Myanmar, the 1,500 bedded Mandalay General Hospital is located in Mandalay city. The Mandalay Blood Bank, situated within Mandalay General Hospital, is one of Myanmar’s national blood banks and ranks among the largest blood demand units in the country. The original survey was carried out within the facilities of the Mandalay Blood Bank.

### Study population and study period

The study population comprised first-time blood donors who donated at Mandalay Blood Bank in Mandalay General Hospital, Myanmar, between April and July 2016, aged between 18 and 55.

### Sample size determination

For the original survey, the sample size was determined using the formula n = Z^2^_1-α/2_ p (1-p)/d^2^ where p = 0.5, desired confidence level (Z^2^_1-α/2_) = 95% = 1.96, desired margin of error (d) = 5% = 0.05. The calculated sample size was n = (1.96)^2^ × 0.5 × (1–0.5)/ (0.05)^2^ = 384. Assuming a 10% dropout rate, the total required sample size was adjusted to 422. However, a total of 406 participants were recruited for the study.

### Inclusion and exclusion criteria

The inclusion criteria were first-time blood donors (both voluntary and replacement donors) between the ages of 18 and 55. According to the National Blood Center Myanmar, people in Myanmar who are 18 to 55 years old are eligible to donate blood [10]. Participants who were found unfit for blood donation during the first round of screening as well as those whose health status immediately following a donation was unfit were excluded from this study.

### Sampling technique

The first-time blood donors who came to Mandalay General Hospital to donate blood during business hours and on weekdays were chosen using a convenience sampling technique. This present study included all 406 first-time blood donors from the original survey in the data analysis.

### Data collection tool and procedure

A structured questionnaire administered by an interviewer was used in the original survey. The questionnaire was divided into four sections: (i) background characteristics (including age, gender, education, marital status, occupation, hepatitis B vaccination, and blood group); (ii) knowledge on TTIs; (iii) attitude toward TTIs; and (iv) risk behavior.

The questionnaire regarding knowledge on TTIs was developed based on the previous literatures and the content validity was verified by experts in the original survey, and it was also supported by further recent research [11–15]. A total of 19 items were included, divided into three topics: types of TTIs (four items), mode of transmission (eight items), and prevention of TTIs (seven items). Each question had two options: "yes (scored as one)" and "no (scored as zero)". The classifications of low, moderate, and high knowledge were determined using the mean and standard deviation of a total score that ranged from 0 to 19 [16].

Developed based on prior research, an attitude questionnaire covering the transmission and prevention of TTIs underwent content validity confirmation by experts in the original survey, and it was further supported by recent research [11–14]. Each of the 11 items was assessed on a 5-point Likert scale, with categorizations including Strongly Disagree (1), Disagree (2), Neutral (3), Agree (4), and Strongly Agree (5). Negative statements, constituting four out of the eleven items, were subjected to reverse scoring. Bloom’s cut-off point was applied to distinguish between low (less than 60%), moderate (60–79%), and high (80–100%) classifications based on a total score ranging from 5 to 55 [17, 18].

A comprehensive risk behavior questionnaire was developed, addressing areas such as blood transfusion, blood and sexual contact, caregiving for hepatitis patients, tattoo and body piercing practices, history of contracting or receiving treatment for gonorrhea or syphilis, experience of imprisonment, sharing personal items like toothbrushes or razors, and undergoing acupuncture treatment, based on both previous and recent research [11, 12, 14, 15]. Its content validity for original survey was evaluated by experts. There were 17 items in all, and each had the two options: "yes (score as zero)" and "no (score as one)". The total score had a range of 0 to 17, and the mean was calculated. Scores above the mean value were categorized as “low risk”, whereas scores below the mean value were deemed “high risk”.

Before the day of data collection, the principle investigator, along with two research assistants, meticulously reviewed the questionnaire and conducted through training session on interview techniques, focusing the importance of establishing rapport with participants. Subsequently, data collection took place five days a week, with Saturday and Sunday excluded, until the desired sample size was attained.

### Data analysis

Data analysis was conducted using the IBM SPSS Statistics software package, version 25. Descriptive statistics, employing frequency and percentage, were utilized to analyze background characteristics, knowledge on TTIs, attitude toward TTIs, and risk behavior. Chi-squared test, bivariable logistic regression, and multivariable logistic regression were employed in inferential statistics to look at the determinants (background characteristics, knowledge and attitude) that influence the risk behavior, adjusting for age, occupation, knowledge and attitude. A p-value of less than 0.05 indicated the statistical significance of the association.

### Ethical consideration

Following permission from the Ministry of Health and ethical approval from the protocol board of University of Medicine, Mandalay, Myanmar in February 2016, the original demographic and risk behavior survey regarding TTIs among first-time blood donors was carried out. Permission for data collection was also informed and granted by the administration of Mandalay General Hospital. Authorization to use secondary data in this present study was provided by the Department of Preventive and Social Medicine, University of Medicine, Mandalay on 14 June 2023. During the original survey, participants were briefed on the study’s objectives, benefits, as well as the confidentiality and anonymity of their involvement and data before data collection. Moreover, participants were invited to partake in the study and provided with written informed consent.

In this present study, an application for an exemption from ethical review was submitted to and approved by the Research Ethics Committee of the Faculty of Medicine, Chiang Mai University, Thailand on 8 November 2023 (COE: 0551/2023).

## Results

### Background characteristics of first-time blood donors

Table 1 presents the background characteristics of first-time blood donors. Among the 406 first-time blood donors, 52.9% were under the age of 20, and 53.7% were male. A majority (77.6%) had undergraduate-level education, 84.2% were married, and 55.7% were students. Additionally, 76.8% had not received the hepatitis B vaccine. Blood group distribution was 40.0% for “B”, 33.8% for “O”, 23.4% for “A”, and 8.9% for “AB”.

**Table 1:** Background characteristics of first-time blood donors (N = 406).

Variable	Frequency (N)	Percentage (%)
**Age**		
≤ 20	215	52.9
> 20	191	47.1
**Gender**		
Male	218	53.7
Female	188	46.3
**Education**		
Primary, Middle and High school	87	21.4
Undergraduate	315	77.6
Postgraduate	4	1.0
**Marital Status**		
Single	64	15.8
Married	342	84.2
**Occupation**		
Government employee	20	4.9
Private employee	107	26.4
Student	226	55.7
Other	53	13.0
**Hepatitis B Vaccination**		
No	312	76.8
Yes	94	23.2
**Blood Group**		
A	95	23.4
B	138	40.0
AB	36	8.9
O	137	33.8

### Knowledge, attitude and risk behaviors of first-time blood donors

Table 2 presents knowledge, attitude and risk behaviors of first-time blood donors towards TTIs. Knowledge levels were high in 15.8% of respondents, and 63.6% displayed a high attitude. Notably, 29.3% (N = 119) of first-time donors exhibited high-risk behavior for TTIs.

**Table 2:** Knowledge, attitude and risk behaviors of first-time blood donors (N = 406).

Variable	Frequency (N)	Percentage (%)
**Knowledge**		
Low	44	10.8
Moderate	298	73.4
High	64	15.8
**Attitude**		
Low	5	1.2
Moderate	143	35.2
High	258	63.6
**Risk Behaviors**		
Low risk	287	70.7
High risk	119	29.3

### Association between background characteristics, knowledge, attitude and risk behavior

Table 3 presents the association between background characteristics, knowledge, attitude, and TTIs risk behavior of first-time blood donors. Statistically significant associations with risk behavior were found for age, occupation, and attitude (p-values of 0.050, 0.004, and 0.012, respectively).

**Table 3:** Association between background characteristics, knowledge, attitude and risk behavior (N = 406).

Variable	Risk Behavior		P-value
	High (N = 119)	Low (N = 287)	
	Frequency (Percentage)	Frequency (Percentage)	
**Age**			0.050*
≤ 20	72 (33.5)	143 (66.5)	
> 20	47 (24.6)	144 (75.4)	
**Gender**			0.809
Male	65 (29.8)	153 (70.2)	
Female	54 (28.7)	134 (71.3)	
**Education**			0.782
Primary, Middle and High school	23 (26.4)	64 (73.6)	
Undergraduate	95 (30.2)	220 (69.8)	
Postgraduate	1 (25.0)	3 (75.0)	
**Marital Status**			0.599
Single	17 (26.6)	47 (73.4)	
Married	102 (29.8)	240 (70.2)	
**Occupation**			0.004*
Government employee	7 (35.0)	13 (65.0)	
Private employee	17 (15.9)	90 (84.1)	
Student	75 (33.2)	151 (66.8)	
Other	20 (37.7)	33 (62.3)	
**Hepatitis B Vaccination**			0.510
No	94 (30.1)	218 (69.9)	
Yes	25 (26.6)	69 (73.4)	
**Blood Group**			0.956
A	26 (27.4)	69 (72.6)	
B	42 (30.4)	96 (69.6)	
AB	10 (27.8)	26 (72.2)	
O	41 (29.9)	96 (70.1)	
**Knowledge**			0.115
Low	17 (38.6)	27 (61.4)	
Moderate	79 (26.5)	219 (73.5)	
High	23 (35.9)	41 (64.1)	
**Attitude**			0.012*
Low	4 (80.0)	1 (20.0)	
Moderate	48 (33.6)	95 (66.4)	
High	67 (25.9)	191 (74.1)	

### Factors influencing TTIs risk behavior among first-time blood donors

Table 4 illustrates the factors influencing TTIs risk behavior among first-time blood donors. Results from both bivariable logistic regression and multivariable logistic regression were interpreted. First-time blood donors aged 20 and above exhibited a risk behavior 1.54 times lower (OR = 1.54, 95% CI: 0.99, 2.38) than donors younger than 20 years old (*p* = 0.049). However, this association lost statistical significance in multivariable logistic regression (*p* = 0.214). Knowledge on TTIs did not show any statistical significance with risk behavior. Notably, first-time blood donors with high attitudes exhibited 11.4 times less risk behavior (OR = 11.4, 95% CI: 1.25, 103.83) than those with low attitudes. This association demonstrated statistical significance in bivariable logistic regression (*p* = 0.017) and retained significance in multivariable logistic regression (*p* = 0.012).

**Table 4:** Factors influencing TTIs risk behavior among first-time blood donors (N = 406).

Variable	Risk Behavior		Risk Behavior	
	Crude OR (95% CI)	P-value	Adjusted OR (95% CI)*	P-value
**Age**		0.049*		0.214
≤ 20	1 (ref:)		1 (ref:)	
> 20	1.54 (0.99, 2.38)		1.46 (0.80, 2.66)	
**Gender**		0.809		
Male	1 (ref:)			
Female	1.05 (0.68, 1.62)			
**Education**		0.779		
Primary, Middle and High school	1 (ref:)			
Undergraduate	0.83 (0.49, 1.42)			
Postgraduate	1.08 (0.11, 10.89)			
**Marital Status**		0.596		
Single	1 (ref:)			
Married	0.85 (0.47, 1.55)			
**Occupation**		0.003*		0.074
Government employee	1 (ref:)		1 (ref:)	
Private employee	2.85 (0.99, 8.99)		3.96 (1.13, 11.80)	0.014*
Student	1.08 (0.42, 2.83)		1.73 (0.58, 5.19)	0.327
Other	0.89 (0.30, 2.60)		1.17 (0.38, 3.56)	0.783
**Hepatitis B Vaccination**		0.507		
No	1 (ref:)			
Yes	1.19 (0.70, 1.99)			
**Blood Group**		0.955		
A	1 (ref:)			
B	0.86 (0.48, 1.54)			
AB	0.98 (0.42, 2.31)			
O	0.88 (0.49, 1.58)			
**Knowledge**		0.123		0.705
Low	1 (ref:)		1 (ref:)	
Moderate	1.74 (0.90, 3.37)		1.80 (0.90, 3.61)	0.098
High	1.12 (0.51, 2.48)		1.14 (0.45, 2.92)	0.512
**Attitude**		0.017*		0.012*
Low	1 (ref:)		1 (ref:)	
Moderate	7.92 (0.86, 72.79)		11.91 (1.20, 118.20)	0.034*
High	11.4 (1.25, 103.83)		17.73 (1.80, 175.12)	0.014*

^a^Adjusted with age, occupation, knowledge and attitude

## Discussion

The study of 406 new blood donors revealed common TTIs risk behaviors. While age initially demonstrated an association with lower risk, this correlation lost significance in multivariable logistic regression. Knowledge on TTIs lacked a distinct association with risk behavior. Notably, individuals with high attitude consistently exhibited a significant reduction in risk behavior, a finding supported in both bivariable and multivariable logistic regression analyses.

A favoring result that highlights the potential for a safer blood supply is the substantial proportion of first-time blood donors who display having low risk behavior for TTIs, which is about 70.7%. The fact that the inclusion criteria included first-time blood donors may have contributed to this outcome. Comparing to the age, first-time blood donors who are age over 20 years old exhibited lower risk behavior of TTIs. This may be ascribed to elder persons may possess more health knowledge, a more established understanding of risk behaviors, safe practices, and a greater sense of responsibility. It is important to consider that the present study did not assess the prevalence of TTIs and its trends, even though its findings deviate from an Ethiopian study that indicated a lower prevalence of TTIs among younger blood donors [19]. Variations in study objectives, methods, and population characteristics might lead to discrepancies in findings. The prevalence of TTIs and age are correlated, according to several studies [19–22]. The lack of a prevalence assessment in this present study highlights the necessity for more investigation to improve the knowledge of TTIs in various age groups of blood donors. Furthermore, the results emphasize how important it is to promote blood safety by ongoing education, awareness campaigns, and thorough screening practices for people of all ages.

Male first-time blood donors made up more than half of the sample in this study (53.7%), which is in line with findings from research conducted in Saudi Arabia and Pakistan [23, 24]. This pattern may be due to a reflection of social and cultural factors that affect male blood donor participation. Additionally, it emphasizes the significance of focused education and outreach campaigns to promote a more equal representation of genders in blood donation initiatives [25]. Compared to government employees, private employee reported significantly fewer risk behavior. This might be a result of the intense competition in the workplace, which encourages private employees to prioritize their health and responsibilities. Furthermore, regular screenings for health in the private sector may raise awareness of health concerns, impact on health behavior and encourage safer practices [26]. An important risk for infection and subsequent transmission to recipients is highlighted by the substantial proportion of first-time blood donors (76.8%) who lack the HBV vaccine. The importance of having thorough immunization protocols and rigorous screening procedures to protect both donors and recipients during the blood donation process is highlighted by the present study.

In the present study, 15.8% of first-time blood donors showed high levels of knowledge, which is a relatively small proportion. This observation might be attributed to the fact that first-time donors may have limited exposure to health education on TTIs. This finding differs from that of a study conducted in Qatar on blood donation among university students [27]. The disparity between the two research populations may result from different demographic characteristics, cultural elements, and the targeted focus of health education campaign. The result of the other study on Ethiopians’ knowledge on blood donation at district blood bank supports the finding of this present study that there is low knowledge of blood donation [28]. Despite the fact that knowledge on TTIs is not associated with risk behavior in the present study, knowledge is still important for influencing risk behavior since it allows people to make well-informed decisions regarding their health [29, 30].

More than half of first-time blood donors (63.6%) in this present study displayed a high attitude towards TTIs, and those with high attitudes exhibited significantly lower risk behavior. Other studies have confirmed the high attitude of blood donors, highlighting the importance of attitude in lowering risk in this population [28, 31]. Other studies emphasize the complex interaction between knowledge, attitude, and risk behavior in addition to the association between attitude and risk behavior discovered in this study [31–33]. Particularly, people with higher levels of knowledge tended to have more positive attitude and engage in lower-risk behavior. This emphasizes the crucial role that knowledge plays in forming ethical attitudes and responsible behavior, underlining the need of knowledge in encouraging safe blood donation procedures.

### Limitations of the study

This present study has certain limitations. Due to the study’s single-center data collection at Mandalay Blood Bank, Mandalay General Hospital, its application to larger populations may be limited. It is not feasible to determine causal correlations or assess changes over time due to the cross-sectional nature of the study design. Convenience sampling may result in selection bias, whilst self-reported data may cause recall and social desirability biases. The study was conducted in 2016, thus any changes to society or the healthcare system that occurred since then may not be reflected in the findings. It should be noted, though, that conditions and blood donation activities in Myanmar were pretty much unchanged during this time.

### Strengths of the study

This pioneering study on the determinants of risk behavior for TTIs among first-time blood donors in Myanmar highlights significant strengths. Although the single-center data collection at Mandalay Blood Bank, Mandalay General Hospital could present limitations, measures were implemented to ensure diverse participant representation. The deliberate use of a cross-sectional design provided a focused snapshot of TTIs risk behavior, and the rigorous methodology, including expert validation of a standardized questionnaire and an extensive literature review, bolstered reliability and validity. Despite the study’s 2016 timeframe, stability in conditions and blood donation activities in Myanmar during that period was considered. The multidimensional approach, encompassing TTIs knowledge, attitudes, and socio-demographic factors, transformed potential limitations into interpretative strengths.

## Conclusions

In conclusion, this study provides valuable insights into the determinants of risk behavior for transfusion-transmissible infections among first-time blood donors, emphasizing the roles of attitude and background characteristics. The findings underscore the significance of the low-risk behavior observed among these donors. The results highlight opportunities for improvement through tailored educational initiatives and interventions to enhance blood safety standards. Future research should prioritize investigating the prevalence of TTIs among first-time blood donors and explore the influence of sociocultural factors on risk behavior, along with conducting longitudinal studies to identify emerging trends and guide targeted interventions. This would substantially contribute to establishing a reliable and safe blood supply chain.

## Supporting information

S1 Database (XLSX)
